# Framingham Risk Stratification of Middle-Aged Migraineurs

**DOI:** 10.1155/2020/7351214

**Published:** 2020-02-07

**Authors:** Gyula Bank, Krisztian Kapus, Janos Meszaros, Kornel Mak, Marietta Pohl, Gabriella Pusch, Eva Fejes, Antal Tibold, Gergely Feher

**Affiliations:** ^1^Centre for Occupational Medicine, Medical School, University of Pécs, Pécs, Hungary; ^2^Department of Geriatrics, Bacs-Kiskun District General Hospital, Kecskemét, Hungary; ^3^Department of Neurology, Medical School, University of Pecs, Hungary; ^4^Hospital of Komlo, Komlo, Hungary; ^5^Neurology Ward, Hospital of Szigetvar, Szigetvar, Hungary

## Abstract

*Introduction*. Migraine is a common primary headache disorder involving about 10-15% of the whole population. Several epidemiological and prospective studies showed a link between migraine (especially migraine with aura) and cardio- and cerebrovascular events.

**Objectives:**

We prospectively analyzed the data of vascular event-free middle-aged patients with migraine who were referred to our Headache Clinic between 01/2014 and 01/2018. Framingham 10-year risk were calculated; covariates included in the analysis were age, total cholesterol, HDL cholesterol, systolic blood pressure, antihypertensive medication use, current smoking, and diabetes status.

**Results:**

Total of 1037 patients were screened and 221 were selected, 161 were women (mean age 55.5 ± 5.2 years) and 60 were men (mean age 56 ± 6 years). 25 patients (11.3%) were labelled as having low risk, 162 patients (73.3%) had moderate risk, and 34 patients (15.4%) had high or very high risk. Blood pressure and lipid targets were reached in 73% and in 49% in the moderate risk and in 53% and 12% in the high risk/very high risk groups, respectively. Migraine with aura (MA) was associated significantly higher cardiovascular risk profile compared with migraine without aura (MO). About one-third of our nondiabetic patients had fasting blood glucose above the normal levels. 24 patients (mean age 60 ± 4.9 years) were diabetic. Mean blood pressure was 149/85 Hgmm, mean choleterol was 5.11 mmol/l, and mean LDL was 2.93 mmol/l in this subgroup, respectively, which do not fall within the recommended targets.

**Conclusion:**

Our article draws attention to the higher cardiovascular risk profile of middle-aged migraineurs and highlights the deficiency of primary prevention. Pain physicians must be aware of the cardiovascular aspects of migraine and holistic approach is required instead of focusing only on pain and pain relief.

## 1. Introduction

Migraine is a common primary headache disorder involving about 10-15% of the whole population, which means that more than one billion individuals are estimated to have migraine [1] It is the third leading cause of disability, and characterized by severe, pulsating, mostly unilateral (often secondarily generalized) headache accompanied by vomiting, nausea, and autonomic dysfunctions (migraine without aura (MO)), sometimes preceded by neurological symptoms (most often visual, but also including sensory symptoms, paresis, or brainstem signs, so-called migraine with aura (MA)) [1, 2].

Several epidemiological and prospective studies showed a link between migraine (especially MA) and cardio- and cerebrovascular events [2–5]. A recent updated meta-analysis including more than one million individuals showed a significant association between migraine and vascular diseases mostly driven by the higher risk of stroke and myocardial infarction [6].

The linking mechanisms seem to be complex and not fully elucidated [3]. Cortical spreading depression (which is the main trigger of aura) is associated with cerebral hypoperfusion, endothelial dysfunction, and the release of free radicals potentially leading to white matter hyperintensities and stroke-like lesions in the posterior circulation, which can be the predecessors of stroke syndromes [3, 6–8]. Migraineurs usually have positive family anamnesis, sedentary lifestyle with obesity and metabolic syndrome, significant subclinical markers of atherosclerosis including higher levels of platelet aggregation, von Willebrand factor and higher prevalence of hypercoagulable states, and more frequent major cardiovascular risk factors [3, 6–9].

To improve the management of patients with headache, the Hungarian Headache Society established 29 Headache Centers accepting referrals from general practitioners (and other medical professioners) or from neurologists not specialized in headache [10].

The Hospital of Szigetvar is a primary hospital covering more than 70000 patients in Southwest Hungary [10]. Our outpatient headache clinic is the “youngest” in our country, launched in 2014.

Based on our knowledge and literature research, only relatively few studies identified patients with high cardiovascular risk and we have no data with regard to real-life Framigham score-based management of event-free migraineurs (including medications and reaching target metabolic parameters), so here we present a modified Framigham score-based evaluation of vascular event-free middle-aged migraineurs referred to our headache clinic.

## 2. Patients and Methods

### 2.1. Patients

We prospectively analyzed the data of patients with headache who were referred to our Headache Clinic between 01/2014 and 01/2018. Headaches were classified based on the IHS criteria [11].

*Inclusion criteria* included a definitive diagnosis of migraine (both MO and MA, and both episodic and chronic forms), both sexes, aged between 45 and 65 years, and having routine blood test results, including total and LDL cholesterol values (TC and LDL-c, in mg/dl) obtained.

*Patients were excluded* if they presented with other headaches than migraine (including medication-overuse headache), having younger than 45 and older than 65 years, the presence of vascular events (stroke, myocardial infarction, angina syndromes, and peripheral arterial disease), having severe uncompensated concomittant diseases (for example, uncompensated endocrine disorder; cholestasis; renal, infectious, and liver disease; and current neoplasia).

### 2.2. Cardiovascular Risk Factors and Framingham Score

*Cardiovascular risk factors* of relevance to this study included smoking habit, diabetes mellitus, hypertension, and dyslipidaemia. A concomitant medication history was taken with respect to use of beta-adrenoreceptor blockers, angiotensin-converting enzyme (ACE) inhibitors, angiotensin (AT) II receptor blockers, and statins.

*Hypertension* was diagnosed in patients with elevated blood pressure values (>140/90 Hgmm, measured twice in a resting position) and in subjects taking antihypertensive therapy. *Dyslipidaemia* was defined as treated with medication or according to the ESC guidelines [12]. *Diabetes* was defined as fasting glucose >130 mg/dl or being on antidiabetic medication according to the ESC guidelines [13].

Estimated 10-year global cardiovascular risk was calculated by the *modified Framingham Risk Score* based on the publication of Agostino et al. [14]. Covariates included in the analysis were age, total cholesterol, HDL cholesterol, systolic blood pressure, antihypertensive medication use, current smoking, and diabetes status [14].

(1) $$\begin{array}{c} \text{RiskFactors}=\left(\ln\left(\text{Age}\right)*\text{AgeFactor}\right)+\left(\ln\left(\text{TotalChol}\right)*\text{TotalCholFactor}\right)+\left(\ln\left(\text{HDLChol}\right)*\text{HDLCholFactor}\right)+\left(\ln\left(\text{SysBP}\right)*\text{SysBPFactor}\right)+\text{Cig}+\text{DM}–\text{AvgRisk}\\ \text{Risk}=100*\left(1-{\text{RiskPeriodFactor}}^{\text{e}\left(\text{RiskFactors}\right)}\right) \end{array}$$

Patients were classified into three Framigham score groups based on the recent ESC guideline criteria: low risk, intermediate risk, and high/very high risk [15].

Data were evaluated as means ± SD (standard deviation) by Student's *t*-test and the chi square test.

## 3. Results

A total of 1037 patients were screened and 221 were selected accordingly to the inclusion/exclusion criteria: 161 women (mean age 55.5 ± 5.2 years) and 60 men (mean age 56 ± 6 years). Baseline characteristics can be seen in Table 1.

**Figure 1:**
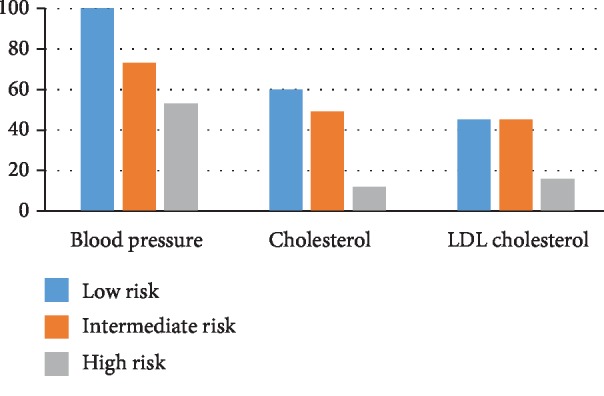
Reaching of target metabolic parameters in the different study groups.

Only 25 patients (11.3%) were classified as having low cardiovascular risk, all of them were women (mean age 49.67 ± 4.15 years). Blood pressure was below 140/90 Hgmm in all cases; mean cholesterol and LDL levels were 5.4 and 3.4 mmol/l, respectively. They had no diabetes, but 8 patients (32%) had fasting glucose above the normal levels (>5.6 mmol/l) (Figure 1).

Vast majority of our headache patients were classified as having moderate cardiovascular risk. This subgroup included 162 patients (73.3%) (mean age 56.23 ± 5.1 years), 26 men (mean age 54.6 ± 6.8 years) and 136 women (mean age 56.6 ± 4.7 years). Blood pressure was above 140/90 Hgmm in 44 patients (27%), and 84 patients had lipid levels above the recommended targets (51%). Eight patients (3%) were diabetic and 40 patients (26%) had fasting glucose above the normal levels (Figure 1).

34 patients (15.4%) were classified as having high/very high cardiovascular risk. This group consisted of 34 men (mean age 57.1 ± 5 years) and 12 patients were diabetic (35%). Blood pressure was above the recommended level in 16 patients (47%), and 30 patients (88%) did not reach the target lipid levels. Furthermore, 8 patients (36%) had fasting glucose above the normal range (Figure 1).

MA patients were younger (53.8 ± 6.4 vs. 56.2 ± 5 years, *p* = 0.005) than MO patients and all but one were female (114 females and 59 males in the MO group) (*p* < 0.001). Despite younger age and female predominance, MA patients had significantly worse modified Framingham scores than MO patients, due to the higher rate of hypertension (75 vs 53.2%, *p* = 0.006), diabetes (25 vs 11.6%, *p* = 0.019), and higher cholesterol levels (5.77 vs. 5.21 mmol/l, *p* = 0.02) (Table 2).

24 patients (mean age 60 ± 4.9 years) were diabetic. Mean blood pressure was 149/85 Hgmm, mean choleterol was 5.11 mmol/l, and mean LDL was 2.93 mmol/l respectively, which do not fall within the recommended targets.

## 4. Discussion

Migraine is a chronic disorder and amongst the leading causes of disability. It affects a large proportion of the population, with female and middle-aged predominance, resulting in significant impact both on the individual and the society. Migraine attacks have a complex pathophysiology involving both neuronal and vascular mechanisms [16]. These mechanisms, particularly those related to inflammation, oxidative stress, and endothelial dysfunction, have raised the possible association between migraine and vascular events [4].

As cardio- and cerebrovascular diseases are the leading causes of death and disability worldwide, the role of screening and prevention is extremely important. Numerous risk assessment systems are widely available (including free online calculators), one of them is the Framingham risk score. To screen our patients, we used a Framingham algorithm that was developed to identify persons at high risk of atherosclerotic CVD, CHD, stroke, intermittent claudication, and heart failure published by D'Agostino et al. [14].

Based on our result, only approximately ten percent of middle-aged migraineurs could be classified as having low cardiovascular risk profile; vast majority have moderate and about 15 percent of our patients have high or very high cardiovascular risk. These results are significantly different to previous Hungarian screening programs.

The Budakalasz study was a population-based screening programme in the Central Hungarian region including 2420 people (mean age 54.8 ± 14.8 years). Event-free patients could be categorized as having low risk (47.6%), moderate risk (41.4%), and high/very high risk (12%) which is parallel to the findings of previous studies [17–19].

In the largest questionnaire-based Hungarian study published in 2003, covering more than 80000 people showed that event-free patients can be categorized as having low risk (62%), moderate risk (27.7%), and high risk (9.7%) [20].

Based on the results of previous studies, the rate of low-risk patients can be approximately 50% in age- and sex-matched middle-aged persons based on Hungarian epidemiological data (in contrast, 11.3% in our population), and the rate of moderate and high/very high-risk patients can be significantly lower in the general population, so our migraineurs have higher cardiovascular risk in general [17–20].

Framigham score elevation was more pronounced in patients with MA comparing to MO. This is in parallel with the findings of the Hunt study [8]. They supposed different mechanisms apart from traditional risk factors in the background of the elevated risk in MA. This is in contrast to our findings; patients with MA all but one were females, younger, and had significantly worse cardiovascular profile (including smoking habits, rate of hypertension and diabetes, and elevated cholesterol levels) overriding the protective effect of female gender and younger age.

Increased Framigham score may arise from the findings that migraineurs usually have positive family anamnesis, sedentary lifestyles with obesity and metabolic syndrome, significant subclinical markers of atherosclerosis including higher levels of platelet aggregation, von Willebrand factor, and higher prevalence of hypercoagulable states [3–6]. On the other hand, a recent study showed a positive correlation between blood lipids and migraine intensity; migraine prophylactic therapy can lead to significant reduction of these parameters [21].

Furthermore, about one-third of our patients had fasting glucose levels exceeding the normal range. Apart from a sedentary lifestyle and metabolic syndrome, increased fasting neuropeptide Y levels in migraine can also be responsible for insulin resistance (and subsequent glucose metabolism changes) by specific alterations in energy intake and activation of the sympathoadrenal system [22].

The entire process in the background of increased vascular risk is not entirely clear, but strong evidence suggests the association of migraine and unfavourable cardiovascular outcome [4–6].

Our study is the first to show the deficiency of primary prevention in middle-aged migraineurs, especially those with high/very high cardiovascular risk. Many patients did not reach the target ESC recommendation levels in blood pressure and metabolic parameters. In a cardiovascular point of view, maybe diabetic migraineurs were the most neglected subgroup in our population.

In general, our article draws attention to the higher cardiovascular risk of middle-aged migraineurs and highlights the deficiency of primary prevention. Pain physicians must be aware of the cardiovascular aspects of migraine, and holistic approach is required instead of focusing only on pain and pain relief.

Finally, our article has some limitations. It was a prospective, single-center study in nature. Secondly, a referral bias was inherently present in our study, does not reflect normal age and gender distribution of migraineurs, and patients with long-standing and disabling headaches were referred as it was conducted at a specialty care center; therefore, it may not be representative of migraineurs in the general population. We had no detailed information on the use of specific drugs for migraine that might be associated with unfavourable side effects (for example, hypertension in chronic NSAID users). And finally, follow-up was not carried out.

## Figures and Tables

**Table 1:** Baseline characteristics of the study population.

Study population	221 (100%)
Mean age	55.61 ± 5.4 years
Males	60 (27.1%)
Females	161 (62.9%)
Migraine	173 (78.3%)
Migraine with aura	48 (21.7%)
Smoking	60 (27%)
Dyslipidaemia	40 (18%)
Hypertension	128 (58%)
Diabetes	24 (11%)
ACE inhibitors	80 (36.2%)
ARBs	28 (12.7%)
Statins	40 (18%)
Beta-blockers	68 (31%)
Blood pressure (Hgmm)	137/83
Cholesterol (mmol/l)	5.36
LDL (mmol/l)	3.12
GFR (ml/min)	74.23

(ACE inhibitors: angiotensin-converting-enzyme inhibitors; ARBs: angiotensin receptor blockers; LDL: low-density lipoprotein; GFR: glomerular filtration rate).

**Table 2:** Comparison of migraine patients with and without aura.

	Migraine without aura	Migraine with aura	*p* values
Study population	173	48	n.a.
Mean age	56.2 ± 5 years	53.8 ± 6.4 years	0.005
Males	59 (34.1%)	1 (2%)	<0.001
Females	114 (65.9%)	47 (98%)	<0.001
Smoking	48 (27.7%)	12 (25%)	0.14
Dyslipidaemia	31 (18%)	9 (18.7%)	0.89
Hypertension	92 (53.2%)	36 (75%)	0.006
Diabetes	20 (11.6%)	12 (25%)	0.019
ACE inhibitors	56 (32.3%)	24 (50%)	0.024
ARBs	20 (11.6%)	8 (16.7%)	0.34
Statins	36 (20.1%)	4 (8.3%)	0.04
Beta-blockers	48 (27.7%)	20 (41.7%)	0.06
Blood pressure (Hgmm)	137/84	138/82	0.23
Cholesterol (mmol/l)	5.21	5.77	0.02
LDL (mmol/l)	3.13	3.1	0.83
GFR (ml/min)	73.95	74	0.77

(ACE inhibitors: angiotensin-converting-enzyme inhibitors; ARBs: angiotensin receptor blockers; LDL: low-density lipoprotein; GFR: glomerular filtration rate).

## Data Availability

The dataset supporting the conclusions of this article is available on request to the corresponding author.
